# Phase-matching-induced near-chirp-free solitons in normal-dispersion fiber lasers

**DOI:** 10.1038/s41377-022-00713-y

**Published:** 2022-01-25

**Authors:** Dong Mao, Zhiwen He, Yusong Zhang, Yueqing Du, Chao Zeng, Ling Yun, Zhichao Luo, Tijian Li, Zhipei Sun, Jianlin Zhao

**Affiliations:** 1 Key Laboratory of Light Field Manipulation and Information Acquisition, Ministry of Industry and Information Technology, School of Physical Science and Technology, Northwestern Polytechnical University, 710129 Xi’an, China; 2 College of Electronic and Optical Engineering & College of Microelectronics, Nanjing University of Posts and Telecommunications, 210046 Nanjing, China; 3 Guangdong Provincial Key Laboratory of Nanophotonic Functional Materials and Devices & Guangzhou Key Laboratory for Special Fiber Photonic Devices and Applications, South China Normal University, 510006 Guangzhou, China; 4 Department of Electronics and Nanoengineering and QTF Centre of Excellence, Aalto University, Aalto, Finland

**Keywords:** Solitons, Mode-locked lasers

## Abstract

Direct generation of chirp-free solitons without external compression in normal-dispersion fiber lasers is a long-term challenge in ultrafast optics. We demonstrate near-chirp-free solitons with distinct spectral sidebands in normal-dispersion hybrid-structure fiber lasers containing a few meters of polarization-maintaining fiber. The bandwidth and duration of the typical mode-locked pulse are 0.74 nm and 1.95 ps, respectively, giving the time-bandwidth product of 0.41 and confirming the near-chirp-free property. Numerical results and theoretical analyses fully reproduce and interpret the experimental observations, and show that the fiber birefringence, normal-dispersion, and nonlinear effect follow a phase-matching principle, enabling the formation of the near-chirp-free soliton. Specifically, the phase-matching effect confines the spectrum broadened by self-phase modulation and the saturable absorption effect slims the pulse stretched by normal dispersion. Such pulse is termed as birefringence-managed soliton because its two orthogonal-polarized components propagate in an unsymmetrical “X” manner inside the polarization-maintaining fiber, partially compensating the group delay difference induced by the chromatic dispersion and resulting in the self-consistent evolution. The property and formation mechanism of birefringence-managed soliton fundamentally differ from other types of pulses in mode-locked fiber lasers, which will open new research branches in laser physics, soliton mathematics, and their related applications.

## Abstract (web summary)

The fiber birefringence, chromatic dispersion, and nonlinear effect follow a phase-matching principle, enabling the formation of birefringence-managed solitons in normal-dispersion fiber lasers containing a section of polarization-maintaining fiber.

## Graphical abstract

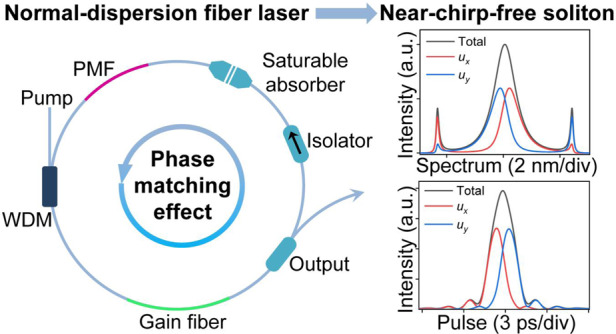

## Introduction

Soliton initially refers to a special type of wavepacket that is capable of propagating undistorted over long distance, which has been broadly discovered in fields of plasma physics^1,2^, fluid dynamics^3–5^, Bose-Einstein condensates^6–8^, and optical networks^9^. In the context of fiber optics, temporal solitons were recommended for optical communications since 1973^10^, and experimentally observed in single-mode fiber (SMF) by several groups^11–13^. After that, various optical solitons were achieved in mode-locked fiber lasers that took into account not only the dispersion and Kerr nonlinearity but also the laser gain and loss^14–18^. The formation of soliton in fiber/fiber laser arises from the interplay between the anomalous dispersion and self-phase modulation effect, enabling the chirp-free feature and Sech^2^ intensity profile. Attributing to periodic perturbations such as amplification and loss, the soliton maintains its nature by dispersing a part of energy displayed as Kelly sidebands^19,20^.

By setting the cavity dispersion into the normal regime, fiber lasers support self-similar pulses and dissipative solitons with the assistance of saturable absorber (SA). Realization of self-similar pulses depends crucially on the self-similar amplification in gain fiber, enabling the parabolic spectral and temporal profiles^21,22^. In contrast, dissipative solitons originate from the mutual-interaction of the nonlinear gain, loss, normal dispersion, and fiber nonlinearity, taking on a variety of spectral/temporal shapes^23–26^. Attributing to the coaction of normal dispersion and SPM effect, both types of pulses are highly chirped and direct generation of chirp-free soliton in normal-dispersion fiber lasers remains a long-term challenge for decades.

Beside the SPM and dispersion effects, the cross-phase modulation (XPM) should be included to describe pulse propagation in birefringent fibers or fiber lasers. Based on the aforementioned effects, polarization-locked vector solitons^27^ and group-velocity locked vector solitons^28^ have been achieved in low-birefringent fibers or fiber lasers. For hybrid-structure lasers comprising low-birefringent SMFs and high-birefringent polarization-maintaining fibers (PMFs), the polarization orientation and cavity effect should be considered particularly. In previous schemes, the PMF usually combines with a polarizer and forms a Lyot filter to control the wavelength and bandwidth of scalar solitons^29,30^. The case becomes much complex without using polarizers or other polarization-sensitive components, which however has received less attention.

Here, we demonstrate near-chirp-free solitons with spectral sidebands in an all-normal-dispersion ytterbium-doped fiber (YDF) laser comprising a section of PMF. The soliton has a bandwidth and duration of 0.74 nm and 1.95 ps respectively, giving the time-bandwidth product of 0.41. To meet the periodic boundary condition of the fiber laser, the pulse follows a phase-matching principle that incorporates the birefringence, normal-dispersion, and nonlinear effects. Simulation results and analytic solutions fully reveal the soliton formation mechanism that the phase-matching effect confines the spectrum broadened by self-phase modulation and the saturable absorption effect slims the pulse stretched by normal dispersion. Such a unique type of pulse is termed as birefringence-managed soliton (BMS) to highlight the role of PMF for implementing the self-consistent evolution. Our work fills the gap for directly generating near-chirp-free solitons in normal-dispersion fiber lasers without external compression, which is quite attractive for wavelength band lower than 1.3 μm where the silica fiber dispersion is typically normal.

## Results

### Principle and simulation/experiment results of BMSs

**Fig. 1:**
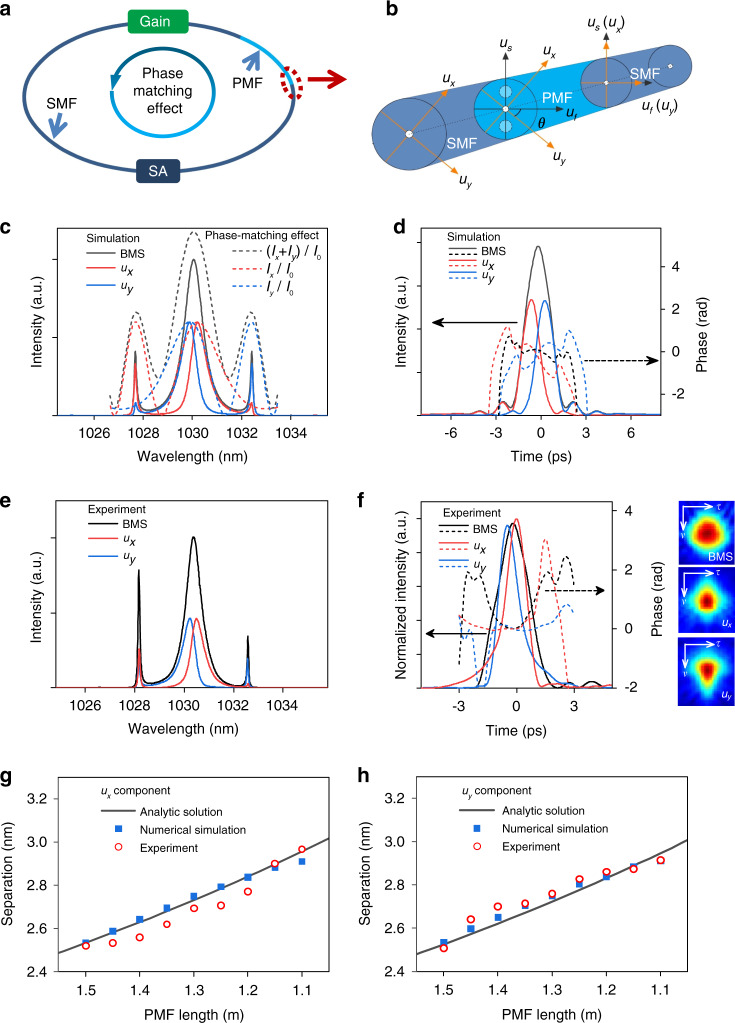
Principle and simulation/experiment results of BMS in YDF lasers. **a** Schematic diagram of normal-dispersion fiber lasers. **b** Coupling behavior of pulse from SMF to PMF. **c** Simulated BMS spectrum and principle of phase-matching effect. **d** Temporal profile and phase. **e** Measured spectra. **f** Retrieved temporal profile and phase from FROG spectrogram. Evolution of sideband separations with PMF length: **g** *u*_*x*_ and **h** *u*_*y*_ components. SMF single-mode fiber, PMF polarization-maintaining fiber, SA saturable absorber.

The configuration of the laser cavity is shown in Fig. 1a, which includes a section of gain fiber, a SA, and a few meters of PMF. The other fibers and pigtails of fiber components are the standard low-birefringent SMFs. A polarization-insensitive isolator ensures the unidirectional propagation of pulse. We construct an all-normal-dispersion YDF laser containing 1.5 m PMF as a typical research platform. The total length and net-dispersion of the fiber laser are ~9 m and 0.214 ps^2^ respectively (See details in Supplementary section I).

When the pulse circulates in the cavity, the coupling behavior from SMF to PMF must be taken into account, as outlined in Fig. 1b. For a certain polarization orientation *θ* (i.e., the angle between *y*-polarized component *u*_*y*_ and fast axis of PMF), two orthogonal-polarized components along the slow (*u*_*s*_) and fast (*u*_*f*_) axes of PMF can be expressed by *u*_*x*_ and *u*_*y*_ that along SMF:

1 $$\begin{array}{l}u_s = u_x\cos \theta - u_y\sin \theta \\ u_f = u_x\sin \theta + u_y\cos \theta \end{array}$$

At the output terminal of PMF, without loss of generality, *u*_*s*_ and *u*_*f*_ components are assumed to translate into the *u*_*x*_ and *u*_*y*_ components along SMF separately. Considering the mode coupling theory described in Eq. (1), we simulate the pulse evolution in the YDF laser according to the experiment parameters. The propagation of pulses in SMF and PMF follows the coupled Ginzburg-Landau equations that take into account the dispersion, birefringence, SPM, XPM, gain, and loss. Equation (2) are numerically solved with the split-step Fourier approach^31–33^ (See details in “Methods”):

2 $$\begin{array}{ll}\frac{{\partial u_x}}{{\partial z}} = - i\beta u_x + \delta \frac{{\partial u_x}}{{\partial t}} - i\frac{{k_2}}{2}\frac{{\partial ^2u_x}}{{\partial t^2}} + i\gamma \left( {\left| {u_x} \right|^2 + \frac{2}{3}\left| {u_y} \right|^2} \right)u_x \\ \qquad \quad + \,\frac{{i\gamma u_y^2u_x^ \ast }}{3} + \frac{{(g - l)}}{2}u_x + \frac{g}{{2{\Omega}_g^2}}\frac{{\partial ^2u_x}}{{\partial t^2}}\\ \frac{{\partial u_y}}{{\partial z}} = i\beta u_y - \delta \frac{{\partial u_y}}{{\partial t}} - i\frac{{k_2}}{2}\frac{{\partial ^2u_y}}{{\partial t^2}} + i\gamma \left( {\left| {u_y} \right|^2 + \frac{2}{3}\left| {u_x} \right|^2} \right) u_y \\ \qquad \quad+ \,\frac{{i\gamma u_x^2u_y^ \ast }}{3} + \frac{{(g - l)}}{2}u_y + \frac{g}{{2{\Omega}_g^2}}\frac{{\partial ^2u_y}}{{\partial t^2}}\end{array}$$

In our simulation, near-chirp-free BMSs can be formed when *θ* ranges from 0.1 π to 0.4 π and giant-chirp dissipative solitons are achieved for *θ* of 0 or 0.5 π, which coincide with the experimental results that BMSs are observed in most cases while the dissipative solitons are obtained at several special states (See details in Supplementary Section II). As a typical example, we first compare the simulated BMS (Fig. 1c, d) with that of the measured counterpart (Figs. 1e and 1f) for PMF length of 1.5 m and *θ* of π/4. The saturation energy is set as 33 pJ, comparable with that of the pulse energy of 38 pJ obtained in the experiment.

As shown in Fig. 1c, e, the spectra of simulated and measured BMSs show the quasi-Sech^2^ profiles with sharp sidebands. It is worth noting that, two orthogonal-polarized components exhibit mirrored spectral profiles with asymmetric sidebands. Taking the *u*_*x*_ component as an example (red solid curve), the stronger sideband is farer to its central wavelength than the weaker one. Such sidebands are formed in a normal-dispersion regime, fundamentally differs from the symmetrically distributed Kelly sidebands that only exist in anomalous-dispersion regime. The formation mechanism of the sidebands will be elaborated later with the phase-matching theory.

The temporal profiles and phases of the BMS, *u*_*x*_ and *u*_*y*_ components are measured by a frequency-resolved optical gating (FROG), as illustrated in Fig. 1f. One can observe that, two orthogonal-polarized components exhibit asymmetric quasi-Lorentz profiles with their phases approaching to constant values at central parts, comparable with the chirp-free pulse reported previously^34–36^. The corresponding autocorrelation traces of BMS is broader than that of two orthogonal-polarized components (Fig. S3 Supplementary section III), also validating the difference of retrieved pulse profiles in Fig. 1f. The measured (simulated) bandwidth and duration are 0.74 nm (0.89 nm) and 1.95 ps (1.95 ps), respectively, giving the time-bandwidth product (TBP) of 0.41 (0.487) with the Sech^2^ fit, which illustrates the near-chirp-free feature of BMS (Fig. 1d). The slight deviation from the Fourier transform limited value is attributed to the spectral and temporal separations between two components. For instance, the bandwidth, duration, and TBP of *u*_*x*_ are 0.62 nm, 1.23 ps, and 0.22 respectively, indicating that each component is near-chirp-free and deviates from the Sech^2^ intensity profile.

Such near-chirp-free BMSs include two unique components with asymmetric spectral sidebands, fundamentally differs from giant-chirp self-similar pulse^37^ and dissipative soliton^23^ reported in the normal-dispersion regime and chirp-free soliton in the anomalous-dispersion regime. It is indicated that the BMS follows a distinct pulse-shaping mechanism than other types of pulses. Informed by solitons formed in anomalous-dispersion regime^20,38^, we propose a phase-matching theory to unveil the underlying physics from mathematical analysis. During the periodic propagation, the pulse experiences SPM, XPM, and perturbations such as the gain/loss and mode coupling, the optical spectrum broadens and disperses new frequencies *ω* with their own velocity. Then, the central frequency *ω*_0_ of each orthogonal-polarized component and the new-emerged frequency *ω* accumulate unequal phases per roundtrip relying on the birefringence, dispersion, and nonlinearity of the cavity. Once new frequencies deviate from the central frequency, the phase difference between them changes continuously from 0, π, to 2 π, as described in Eq. (3) (See detailed derivation in Supplementary section IV).

3 $$\begin{array}{lll}{\Delta}\varphi _x &= &a{\Delta}\omega _x^2 - b{\Delta}\omega _x - \phi _{nlx}\\ {\Delta}\varphi _y &= & a{\Delta}\omega _y^2 + b{\Delta}\omega _y - \phi _{nly}\\ a &=& \frac{1}{2}\mathop {\sum}\limits_i {\beta _{2i}} L_i \\ b &=& \frac{1}{{2c}}\mathop {\sum}\limits_i {{\Delta}n_i} L_i\end{array}$$

Here *a* and *b* represent the contribution of fiber dispersion and birefringence, respectively. *β*_2_ and Δ*n*_*i*_ represent the dispersion and refractive index difference between two components for fiber with the length of *L*_*i*_. Δ*ω*_*x*/*y*_ is the frequency separation between the central frequency *ω*_0_ and new-emerged *ω* of *u*_*x/y*_ component. *ϕ*_*nl*_ is the nonlinear phase shift accumulated in one roundtrip.

For each orthogonal-polarized component, the new-emerged frequencies between adjacent roundtrip co-propagate inside the cavity and have a phase difference of Δ*φ*_*x*/*y*_ that depends on the Δ*ω*_*x*/*y*_. To give clear demonstration, we consider the interference of two lights with intensity *I*_0_ and phase difference Δ*φ*_*x*/*y*_, and obtain the phase-matching relation for each orthogonal-polarized component, as expressed in Eq. (4) (the details are given in Supplementary section V):

4 $$\begin{array}{l}I_x({\Delta}\omega_{x})/I_0 = 2(1 + \cos{\Delta}\varphi_{x})\\ I_y({\Delta}\omega_{y})/I_0 = 2(1 + \cos {\Delta}\varphi_{y})\end{array}$$

Consequently, with the increase of Δ*ω*_*x*/*y*_, the new-emerged frequencies between adjacent roundtrips interfere constructively, destructively, and constructively, as denoted by the dotted curves in Fig. 1c.

The spectral profile, sideband intensity and position of each component are asymmetric, which differs from conventional soliton with symmetrical spectrum and sideband^20^. Such unique spectra result from the unsymmetrical birefringence-related phase-matching effect that the stronger sidebands appear for the phase difference of 2 π, as described in Eq. (4) and Fig. 1c. In the analytical calculation, the sideband separations are unequal and one of them is abandoned as it exceeds the gain bandwidth of YDF. Notably, the weaker sideband stems from the mode coupling between two orthogonal-polarized components at the input terminal of PMF per roundtrip. As two components of BMS have different central wavelengths, the stronger sideband is farer to the central wavelength than that of the weaker one for each component. It is indicated that the phase-matching effect not only confines the BMS spectrum but also results in the unsymmetrical spectral sidebands.

By diminishing the PMF length from 1.5 m to 1.1 m with a step of 0.05 m, we record the spectral separations of *u*_*x*_ and *u*_*y*_ components between the stronger sideband and their central wavelengths. The theoretical values Δ*λ*_*x*/*y*_ (calculated from Δ*ω*_*x*/*y*_) based on Eq. (S8) are derived from Eq. (3) by setting Δ*φ*_*x*/*y*_ as 2 π. As shown in Figs. 1g and 1h, both theoretical and simulation results show that the normalized spectral separation increases by clipping the PMF, which agrees with experimental observations and confirm the validity of the phase-matching theory.

**Fig. 2:**
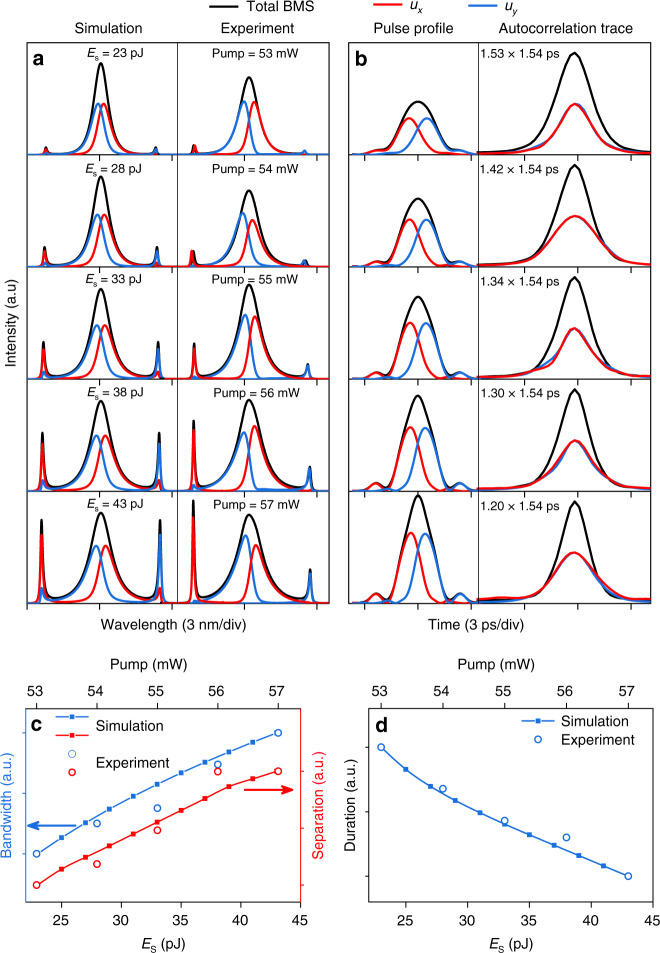
Evolution of BMS with gain saturation energy (*E*_s_) and pump power. **a** Simulated and measured spectra. **b** Simulated pulse profiles and measured autocorrelation traces. **c** Bandwidth of BMS and sideband separation of *u*_*y*_ component versus *E*_s_ and pump power. **d** Pulse duration of BMS versus *E*_s_ and pump power.

With the elevation of pump strength, the simulation and experiment results display the similar evolution behavior (e.g., gradually increased sideband intensity and decreased pulse duration), as illustrated in Fig. 2a, b. To compare the evolution trends clearly, the bandwidth, spectral separation, and pulse duration are normalized using equation (*F*(*x*)−*F*_min_)/(*F*_max_−*F*_min_), as shown in Fig. 2c, d. During this evolution, the sideband intensity increases faster than that of the central spectrum, and two sidelobes appear on the wings of the pulse. Such phenomenon is understood by noting that the spectrum of BMS is strongly limited by the phase-matching effect. For a higher intensity, the spectrum is broadened by nonlinear effects while confined by the phase-matching effect. Then, the redundant energy transfers to the frequencies that the phase-matching condition is satisfied.

As the SPM effect induces a larger nonlinear phase shift *ϕ*_*nl*_^39^, according to the phase-matching condition in Eq. (3), the sideband separation of BMS will enlarge correspondingly with the pump power.

**Fig. 3:**
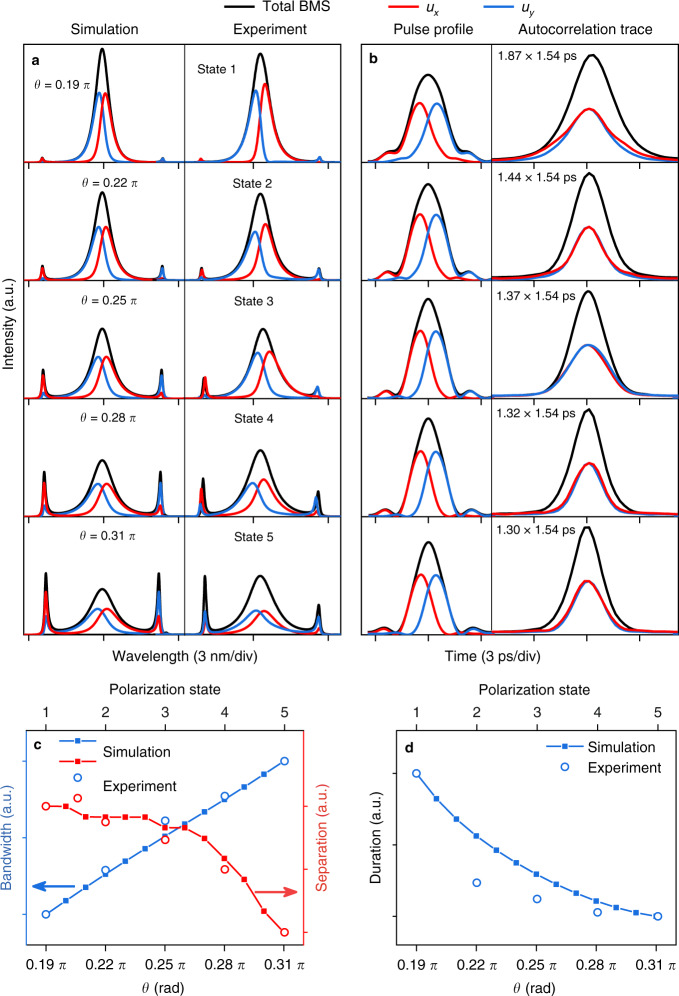
Evolution of BMS with the polarization orientation *θ*. **a** Simulated and measured spectra. **b** Simulated pulse profiles and measured autocorrelation traces. **c** Bandwidth of BMS and sideband separation of *u*_*y*_ component and **d** pulse duration as a function of *θ.*

We further investigate the evolution of BMSs as a function of polarization orientation *θ*, as shown in Fig. 3a, b. In the experiment, the polarization controller placed before the spliced point of SMF and PMF is adopted to change the polarization orientation of pulse. When *θ* increases, a stronger mode coupling occurs between two orthogonal-polarized components, and results in larger spectral sidebands. Such evolution trend is somewhat similar to the well-known Kelly sideband that a stronger perturbation leads to a larger resonant peak, and will be fully elaborated in the latter part. Figure 3c, d shows that the bandwidth increases linearly while the duration decreases slightly due to the near-chirp-free feature of BMS. As part of the energy of the BMS is transferred to the sidebands, the nonlinear phase shift between the central frequency and the sideband decreases slightly. Thus, the spectral separation will reduce according to Eq. (S8) and experimentally confirmed by the red curve in Fig. 3c. As a matter of fact, the BMS can be regarded as a unique group-velocity locked vector soliton arising from co-actions of chromatic dispersion, fiber birefringence, and nonlinearity. By tuning the pump strength or *θ*, the nonlinear effect changes and the central frequencies of two components shift slightly to realize a new balance in the same fiber laser.

For comparisons, we replace the polarization-insensitive isolator by a polarization-sensitive isolator to form a Lyot filter inside the cavity. In this case, the modulation period of Lyot filter is ~1.7 nm for the PMF length of 1.5 m, which is too narrow to achieve mode locking in our fiber laser. By diminishing the PMF length to ~0.4 m, both experimental and simulation results show that, giant-chirp dissipative solitons can be achieved in the fiber laser (See details in Supplementary section VI), which validates that the formation of BMS is dominated by the phase-matching effect rather than the Lyot filtering effect^30^.

Based on the aforementioned results, the formation of the BMS is summarized as follows. In the frequency domain, the spectral broadening caused by the nonlinear effects and periodic perturbations is counterbalanced by the phase-matching effect, results in the limited bandwidth with downshifted and upshifted spectral sidebands. In the time domain, the normal dispersion induced stretching of pulse is compensated by saturable absorption and phase-matching effects, helps to establish the steady-state pulse evolution in a slow-varying style. The PMF length dominates the phase-matching bandwidth, which offers a flexible approach to control the bandwidth and duration of the BMS.

### Buildup process and intracavity evolution of BMSs in YDF laser

**Fig. 4:**
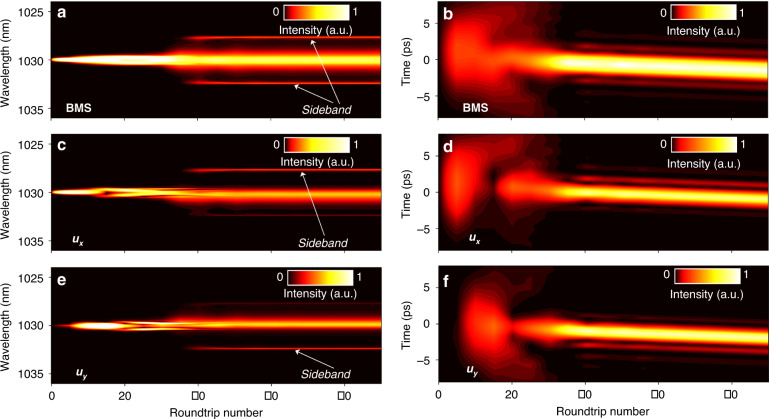
Establishment process of BMS for PMF length of 1.5 m and *θ* of 0.25 π. Simulated spectral and temporal evolutions of (**a**, **b**) BMS, (**c**, **d**) *u*_*x*_ component, and (**e**, **f**) *u*_*y*_ component.

Based on the Ginzburg–Landau equations, we investigate the establishment process of BMS versus roundtrips, as displayed in Fig. 4a, b. The initial signal is a low-intensity noise pulse and *θ* is 0.25 π. During propagation through each fiber component, the light field is multiplied with the corresponding transmission matrix. For the *u*_*x*_ and *u*_*y*_ components shown in Fig. 4c–f, both of them first grow exponentially and then gradually reach a sub-stable state after 10 roundtrips. Subsequently, the bandwidth increases while pulse duration decreases with the roundtrips. Simultaneously, new spectral components emerge due to the coaction of SPM and XPM effects. After that, the noise components are suppressed to support the ultrashort high-intensity pulse, under the well-known saturable absorption effect^31,40–42^. When the quasi-stable state is established at 35 roundtrips, two sharp sidebands appear incrementally on the spectra. The BMS evolve to the self-consistent steady state at 50 roundtrips, in which the two components have unsymmetrical spectral sidebands, as also validated in Figs. 1c and 1d. Apart from the sideband symmetry, the formation process of BMS is similar to its two components.

**Fig. 5:**
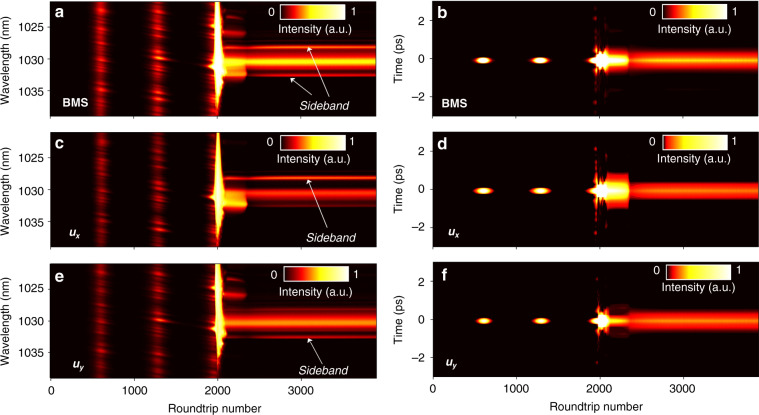
Buildup processes of BMS measured by dispersive Fourier transform technique. Spectral and field autocorrelation evolutions of (**a**, **b**) BMS, (**c**, **d**) *u*_*x*_ component, and (**e**, **f**) *u*_*y*_ component.

With the assistance of the dispersive Fourier transform (DFT) technique capable of mapping the spectrum into time domain^43–46^, we record the buildup process of BMS and its two orthogonal-polarized components for comparison with the simulation counterpart. The left panel of Fig. 5 shows real-time spectral evolutions of BMS and its two components, and the corresponding field autocorrelation trajectories are displayed in the right panel, which is obtained by applying Fourier transform of each single-shot spectrum. As illustrated in Figs. 5a and 5b, after the relaxation oscillation and beating process, the spectrum of BMS broadens drastically and parts of the energy transfer to spectral edge and form the sidebands. After that, the laser reaches the steady state at ~2400th roundtrip. The two orthogonal-polarized components evolve similarly with the BMS while display asymmetric spectral sidebands (Fig. 5c–f), which coincides with the simulation counterpart and further validates the reliability of the proposed model.

**Fig. 6:**
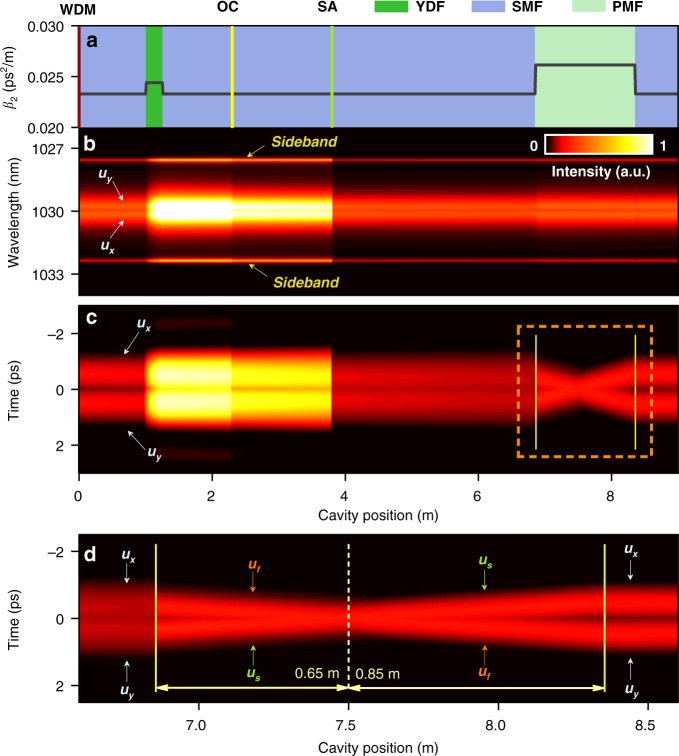
Intracavity evolution of BMS at *θ* = 0.25 π. **a** Cavity position versus fiber components and dispersion parameters. **b** Spectral evolutions of *u*_*x*_ and *u*_*y*_ components. **c** Temporal evolutions of *u*_*x*_ and *u*_*y*_ components. **d** Close-up of the temporal evolution around the PMF region. WDM wavelength division multiplexer, OC optical coupler.

The dynamic evolution of the BMS along the cavity is depicted in Fig. 6. In a single roundtrip, the pulse sequentially propagates through 1 m SMF, 0.25 m YDF, 1.05 m SMF, the optical coupler, 1.5 m SMF, the SA, and 3.05 m SMF. After that, the BMS enters the 1.5 m long PMF, where the *u*_*f*_ component and *u*_*s*_ component propagate in a “X” manner. Such phenomenon results from the birefringence-induced collision and walk-off effect between two orthogonal-polarized components^47^. At the end of the PMF, the BMS reaches the SMF to start the next circulation. A unique feature is that the collision point deviates from the PMF center, which partially counteracts the group delay difference induced by the chromatic dispersion of cavity. For example, the birefringence-induced group delay difference is given as 0.38 ps while that induced by chromatic dispersion is calculated as −0.12 ps. Combined with the saturable absorption effect, the two components centered at different wavelengths finally evolve to the self-consistent state, which is somewhat similar to that of the group-velocity locked vector solitons^48^. The asymmetrical spectrum of each component is a steady-state phenomenon, which differs from the transient unsymmetrical spectrum arising from the periodic coupling between two orthogonal-polarized components^49^.

**Fig. 7:**
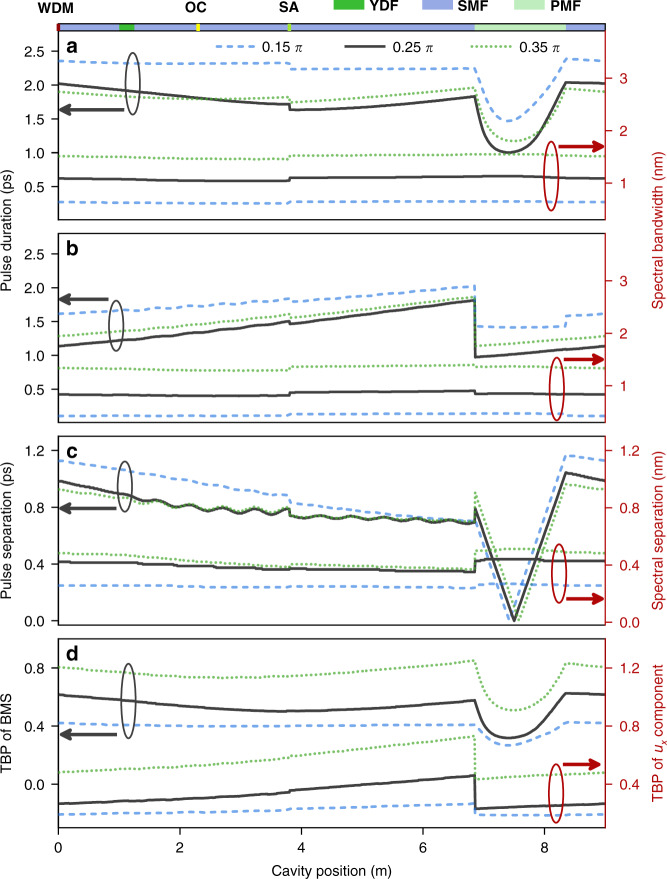
Intracavity evolution of BMS at *θ* = 0.15 π (dash blue line), 0.25 π (black solid line) and 0.35 π (dash olive line). Evolution of spectral bandwidth and pulse duration: **a** BMS and **b** *u*_*x*_ component. **c** Spectral and temporal separation between *u*_*x*_ and *u*_*y*_ components. **d** TBP evolution of BMS and *u*_*x*_ component.

We extract the key pulse parameters of the BMS and its two orthogonal-polarized components during propagation through the cavity for *θ* of 0.15 π, 0.25 π, and 0.35 π. Unlike the BMS in Fig. 7a, the durations of two components increase monotonously along the cavity due to the group-velocity dispersion while decrease abruptly due to the mode coupling in the PMF (Fig. 7b). Such differing evolutions arise from the varied pulse separation between two components, as confirmed by the black curve in Fig. 7c. Interestingly, the bandwidth of each component almost keeps unchanged throughout the cavity, indicating that nonlinear effects play a less important role in the formation of BMS. The TBP of BMS spans from 0.61 at the WDM to 0.31 at middle part of PMF. For comparisons, the TBP of each orthogonal-polarized component enlarges from 0.26 to 0.45 in SMF and YDF, and decreases abruptly to 0.23 at the connection point between SMF and PMF. An interesting phenomenon is that the intensity of sidebands enlarges with *θ* (corresponding to Fig. 3a), which can be explained by noting that a larger *θ* corresponds to a stronger variation of pulse property, as confirmed by Fig. 7a–d. As a matter of fact, the BMS is a ubiquitous phenomenon in normal-dispersion fiber lasers that are independent of the cavity length or mode-locking elements. For example, similar near-chirp-free solitons have also been achieved in normal-dispersion erbium-doped fiber lasers, as shown in Supplementary section VII.

## Discussions and conclusions

In conclusion, we proposed a phase-matching theory to explain the formation of unique near-chirp-free BMS in normal-dispersion SMF-PMF lasers. Based on coupled Ginzburg-Landau equations, we simulated the pulse evolution as a function of PMF length, pump power, and polarization orientation. The theoretical analysis and simulation results fully reproduce and explain the experimental observations. It is indicated that the phase-matching effect confines the spectrum broadened by self-phase modulation and the saturable absorption effect slims the pulse stretched by normal dispersion, enabling the generation of the near-chirp-free soliton. During the evolution, the two orthogonal-polarized components propagate in a “X” manner in the PMF, which partially compensates the group delay dispersion and results in the unique soliton trapping. The BMSs, universally existed in the normal-dispersion regime, are independent of the cavity length or mode-locking elements in the SMF-PMF lasers. Similar BMSs have been achieved in four different normal-dispersion fiber lasers with cavity length ranging from 7.8 m to 25.6 m. Our results confirm that the birefringence can be adopted to shape solitons at the normal-dispersion regime, in contrast to the balance of nonlinear and anomalous-dispersion effects that results in standard solitons.

From an application perspective, the proposed scheme is capable of producing near-chirp-free solitons in normal-dispersion cavities without external compression, especially attractive for wavelength below 1.3 μm where silica fiber dispersion is typically normal^50–52^. Such principle can be extended to propagate near-chirp-free soliton over long distance in normal-dispersion regimes with periodically spaced SMF and PMF. The fiber laser is capable of delivering switchable near-chirp-free BMS and giant-chirp dissipative soliton (See Supplementary Videos S1 and S2 for details), which can also work as a flexible multi-functional pulse source. As the pulse energy of BMS is confined to a limited level, high-order harmonic mode-locking may be achieved in long-cavity normal-dispersion fiber lasers based on the proposed scheme.

## Materials and methods

### Experimental setup

The experiment setup of the fiber laser is presented in Supplementary Fig. S1, in which 0.25 m ytterbium-doped fiber (YDF, Liekki Yb1200-4/125) is pumped by a 976 nm laser diode (LD) through a wavelength division multiplexer (WDM). The 20% port of an optical coupler (OC) is used to output the laser. A polarization-insensitive isolator (PI-ISO) is used to ensure unidirectional propagation of light. A section of polarization-maintaining fiber (PMF, 1.5 m, Nufern PM980-XP) is incorporated into the cavity, and the first polarization controller (PC1) controls the polarization orientation before the PMF. The pigtails of fiber components are SMF with the total length of 7.25 m. A semiconductor saturable absorber (SESA, BATOP) initiates the mode-locking operation. The polarization beam splitter (PBS) placed at the output terminal resolves two orthogonal-polarized components of the BMS. The dispersion parameters *D* for YDF, SMF, and PMF are given as −43, −41, and −46 ps (nm km)^−1^, respectively, and the net cavity dispersion is calculated as 0.214 ps^2^.

### Measurement system

The output lasers are monitored via an optical spectrum analyzer (YOKOGAWA: AQ6370), an autocorrelator (Pulsecheck: USB-150), a radio frequency analyzer (Agilent: E4440A), a 500-MHz oscilloscope (Tektronix: DPO 3054), and a frequency-resolved optical gating (MesaPhotonics: FROGscan) simultaneously. The real-time spectral measurement is performed by 20 km SMF (Corning: SMF-28e) with the accumulated dispersion of ~378 ps^2^, a 5 GHz photodetector (THORLABS: DET09CFC/M), and a 16 GHz high-speed oscilloscope (Tektronix: DPO71604C). The spectral resolution of this DFT system is ~0.53 nm based on the final limitation of DFT method^43^.

### Numerical simulations

For the coupled Ginzburg–Landau equations (Eq. (2)), *u*_*x*_ and *u*_*y*_ represent envelopes of two polarization components of pulse, *t* is the relative time in the moving frame, and *z* is the propagation distance. Δ*n*, 2*β* = 2πΔ*n/λ*, and 2*δ* = 2*βλ/*2πc are the differences of refractive index, wave-number, and inverse group velocity respectively, which relate to the fiber birefringence. *k*_2_ is the group-velocity dispersion, *γ* is the nonlinear coefficient, and *l* represents the loss. *g* = *g*_0_exp(−*E*_p_/*E*_s_) is the saturable gain and Ω_*g*_ is the gain bandwidth, where *E*_p_, *g*_0_, and *E*_s_ are the pulse energy, small-signal gain, and gain saturation energy respectively. The semiconductor saturable absorber is modeled by *T* = 0.45 − *T*_0_/[1 + *P*_(τ)_/*P*_sat_], where *T*_0_ is the modulation depth, *P*_(τ)_ is the instantaneous power and *P*_sat_ is the saturation power. The propagation of the pulse in fiber is modeled by the standard split-step Fourier transform technique.

To match the experiment, the simulation parameters are: *T*_0_ = 0.17, *P*_sat_ = 15 W, *λ* = 1035 nm, *l* = 4.61 × 10^−4^ m^−1^, *γ* = 4.7 × 10^−3^ W^−1^ m^−1^; for SMF, Δ*n* = 0.97 × 10^−6^, *k*_2_ = 23.3 ps^2^ km^−1^; for YDF, *g*_0_ = 64.5 m^−1^, *k*_2_ = 24.4 ps^2^ km^−1^; for PMF, Δ*n* = 3.95×10^−4^, *k*_2_ = 26.1 ps^2^ km^−1^ and other parameters are the same as that of the SMF.

## Supplementary information

Supplemental material

Switching of near-chirp-free BMS and giant-chirp DS in ytterbium-doped fiber laser

Switching of near-chirp-free BMS and giant-chirp DS in erbium-doped fiber laser

## Data Availability

The data that support the plots within this paper and other findings of this study are available from the corresponding authors upon reasonable request.

## Code availability

The code used in this paper is available from the corresponding author upon reasonable request.
