# Supplementary material for: Phase-matching-induced near-chirp-free solitons in normal-dispersion fiber lasers
Source: Light Sci Appl. 2022 Jan 25;11:25. doi: 10.1038/s41377-022-00713-y (PMC8789917; doi:10.1038/s41377-022-00713-y)
Supplement: Supplementary file 1 — Supplemental material [file 41377_2022_713_MOESM1_ESM.docx]

Supplementary Information for

**Phase-matching-induced near-chirp-free solitons in normal-dispersion fiber lasers**

Dong Mao^1^, Zhiwen He^1^, Yusong Zhang^1^, Yueqing Du^1^, Chao Zeng^1^, Ling Yun^2^, Zhichao Luo^3^, Tijian Li^3^, Zhipei Sun^4^*, Jianlin Zhao^1^*

*^1^Key Laboratory of Light Field Manipulation and Information Acquisition, Ministry of Industry and Information Technology, School of Physical Science and Technology, Northwestern Polytechnical University, Xi’an 710129, China*

*^2^College of Electronic and Optical Engineering & College of Microelectronics, Nanjing University of Posts and Telecommunications, Nanjing 210046, China*

*^3^Guangdong Provincial Key Laboratory of Nanophotonic Functional Materials and Devices & Guangzhou Key Laboratory for Special Fiber Photonic Devices and Applications, South China Normal University, Guangzhou 510006, China*

*^4^Department of Electronics and Nanoengineering and QTF Centre of Excellence, Aalto University, Aalto, Finland*

*Corresponding author: [zhipei.sun@aalto.fi](mailto:zhipei.sun@aalto.fi); [jlzhao@nwpu.edu.cn](mailto:jlzhao@nwpu.edu.cn)

**This Supplementary Information consists of the following sections:**

S1. Experimental setup

S2. Simulated and measured DS in the same fiber laser

S3. Autocorrelation traces of BMS and its two orthogonal-polarized components in the YDF laser

S4. Derivation of phase matching principle in SMF-PMF fiber laser

S5. Spectral confinement and sideband generation based on phase matching effect

S6. DS in the YDF laser with a Lyot filter

S7. BMS in the normal-dispersion erbium-doped fiber laser

Supplementary Figures S1-S6

Supplementary Videos S1 and S2

Supplementary References S1-S4

**S1. Experimental setup**





**Fig. S1 Setup of the all-normal-dispersion Yb-doped fiber laser.** LD laser diode, WDM wavelength division multiplexer, YDF ytterbium-doped fiber, OC optical coupler, SESA semiconductor saturable absorber, PI-ISO polarization-insensitive isolator, PC polarization controller, PMF polarization-maintaining fiber, PBS polarization beam splitter.

The experiment setup of the ytterbium-doped fiber laser is presented in Fig. S1, in which the semiconductor saturable absorber initiates the passive mode-locking. The 1.5 m polarization-maintaining fiber (PMF) introduces high birefringence to the fiber laser and the polarization controller (PC1) adjusts the polarization orientation at the input terminal of PMF. Outside the cavity, the pulse is resolved into two orthogonal-polarized components by a polarization beam splitter together with a polarization controller (PC2). The self-starting continuous-wave emission is established at the pump power of 45 mW, while single birefringence-managed soliton (BMS) is obtained for the pump power of 50 to 58 mW.

**S2. Simulated and measured DS in the same fiber laser**

In our simulation, near-chirp-free BMSs can be formed when *θ* ranges from 0.1 π to 0.4 π and giant-chirp dissipative soliton (DS) can be formed when the polarization orientation *θ* is about 0 or 0.5 π. The typical spectral and temporal profiles of BMS are displayed in Figs. 1e and 1f of the manuscript. As demonstrated in Figs. S2a and S2b, the spectrum of DS exhibits typical steep edges and the pulse is strongly chirped, similar with the previous reports^1-3^. The bandwidth and duration are 1.75 nm and 12.14 ps respectively, which give the time-bandwidth product (TBP) of 5.95 and confirm the giant-chirp property of the DS. Through tuning the polarization controller before the PMF, giant-chirp DS operation can also be achieved in the experiment, as plotted in Figs. S2c and S2d. The measured bandwidth and duration are 2.24 nm and 9.26 ps respectively, and TBP is calculated as 5.81 using the Gaussian fit, which agree well with that of the numerical simulations.

Due to the co-action of birefringence and chromatic dispersion of fiber laser, the repetition-rates of DS and BMS change slightly with polarization states, as displayed in Fig. S2e. Because of the limited resolution of oscilloscope, the DS and BMS have the identical pulse-to-pulse separation of ~45.2 ns (Fig. S2f), corresponding to the cavity length of ~9 m.


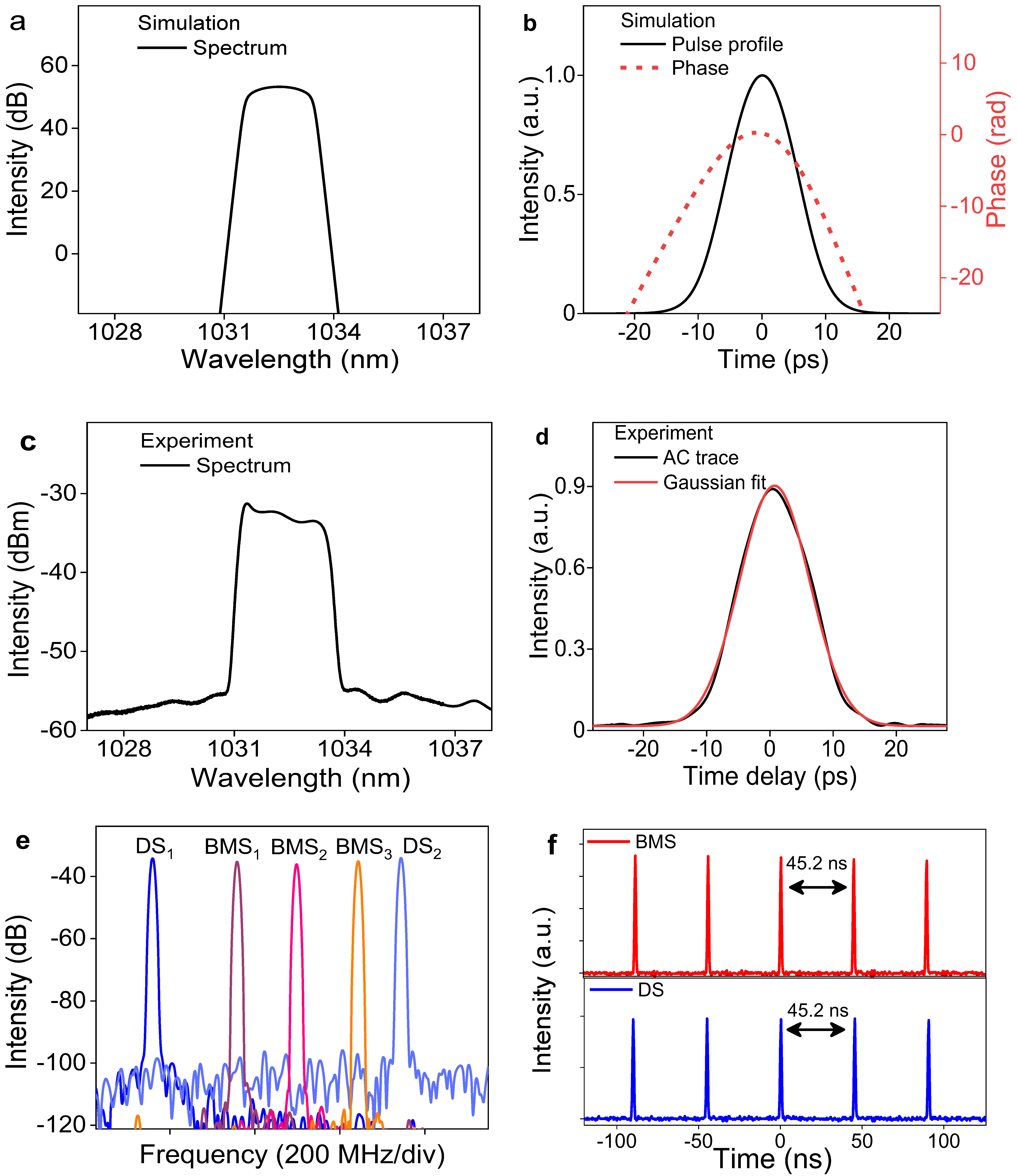


**Fig. S2 Simulated and measured DS in the same fiber laser. a** Simulated spectrum, **b** temporal profile and phase. **c** Measured spectrum, **d** autocorrelation trace and fit curve. **e** Radio frequency spectra of BMS and DS under different polarization states. **f** Pulse trains of BMS and DS.

**S3. Autocorrelation traces of BMS and its two orthogonal-polarized components in the YDF laser**


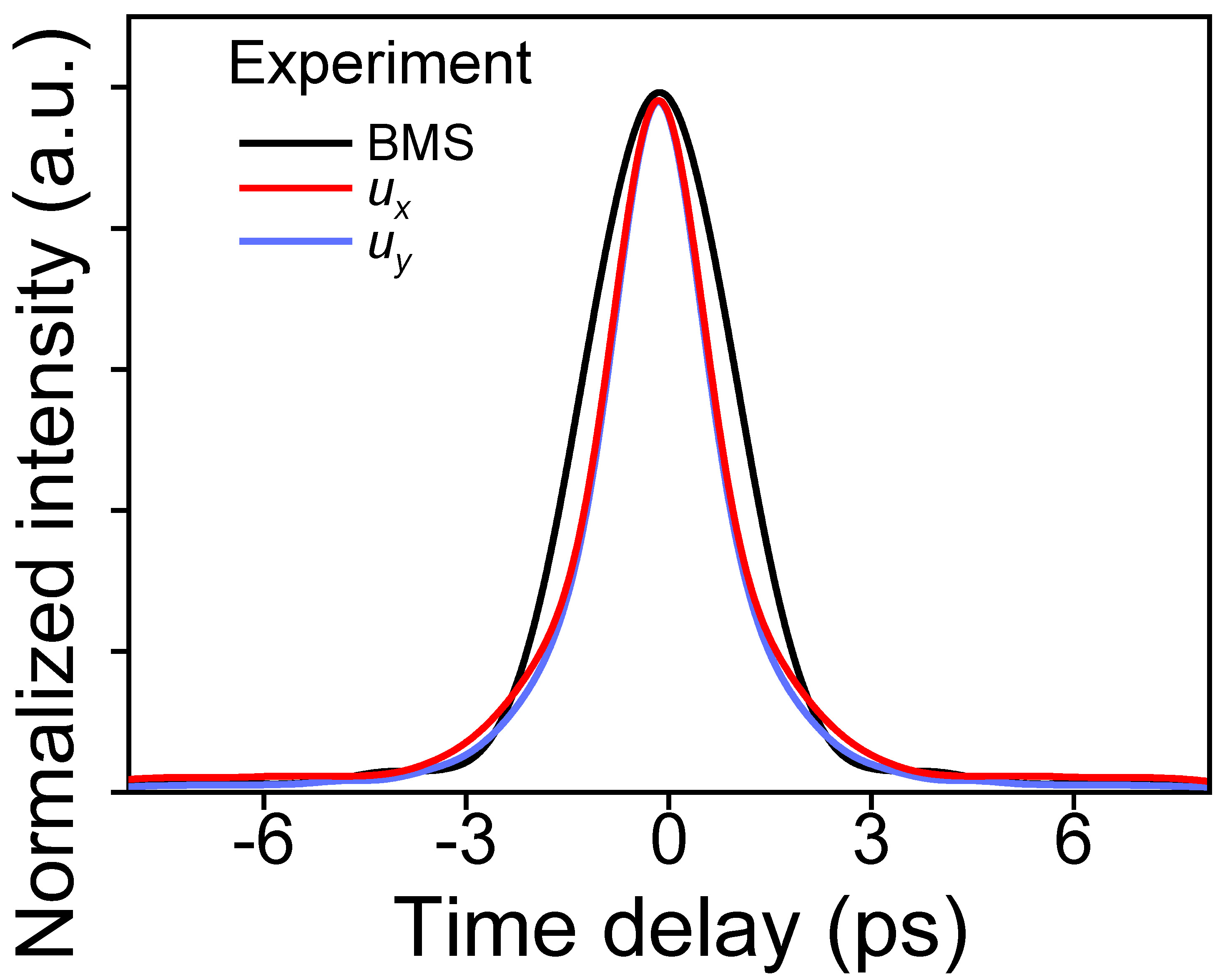


**Fig. S3 Autocorrelation traces of BMS (black curve), *u_x_* component (red curve), and *u_y_* component (blue curve).**

Figure S3 shows that the autocorrelation trace of BMS differs from that of two orthogonal-polarized components, validating the unequal retrieved pulse profiles in Fig. 1f in the manuscript. The durations of BMS, *u_x_*, and *u_y_* components are 1.948 ps, 1.154 ps, and 1.234 ps, respectively. Considering their 3-dB bandwidths of 0.74 nm, 0.58 nm, and 0.62 nm, the TBP is calculated as 0.41, 0.19, 0.22 for BMS, *u_x_*, and *u_y_* components respectively, which further corroborates the near-chirp-free property of such pulses. As the TBP of Sech^2^ pulse is higher than 0.315, we infer that, each component of BMS has an asymmetric quasi-Lorentz profile.

**S4. Derivation of phase matching principle in SMF-PMF fiber laser**

In the hybrid-structure SMF-PMF laser, the spectrum of each orthogonal-polarized component is broadened by the nonlinear effects and emits new-frequency due to various perturbations such as gain, loss, and PMF-induced mode coupling. During propagation in the resonator, the phase difference between frequency *ω* and center frequency *ω*_0_ dominates the interference behavior of frequency emerged at adjacent roundtrips. The mode propagation constant *β* can be expanded at the center frequency *ω*_0_:

$\beta(\omega)=\beta_{0}+\beta_{1}(\omega-\omega_{0})+\frac{1}{2}\beta_{2}(\omega-\omega_{0})^{2}+...$ (S1)

In birefringent fibers, the refractive indices of two orthogonal-polarized components are different, so:

 (S2)

Considering that *β*_0_*_x_* and *β*_0_*_y_* are independent of frequency *ω*, they can be ignored in this case. Since the group velocity dispersion values of two components are almost the same (*β*_2_*_x_*=*β*_2_*_y_*=*β*), the equation can be simplified as:

 (S3)

where *n*_0_ is the average refractive index of fiber, and Δ*n* represents the refractive index difference between two components. After ignoring the constant term *n*_0_/*c* and the higher-order dispersion, the equation can be simplified as:

$\begin{matrix} \beta_{x}(\omega)=-\frac{\Delta n}{2c}(\omega-\omega_{0})+\frac{1}{2}\beta_{2}(\omega-\omega_{0})^{2} \\ \beta_{y}(\omega)=\frac{\Delta n}{2c}(\omega-\omega_{0})+\frac{1}{2}\beta_{2}(\omega-\omega_{0})^{2} \end{matrix}$ (S4)

After propagating through fiber with length of *L*, the relation between the linear phase difference and the frequency difference (Δ*ω=ω-ω*_0_) is:

 (S5)

When the nonlinear phase shift *φ_nl_* is taken into account, the phase differences are:

 (S6)

Here *a* and *b* represent the contribution of fiber dispersion and birefringence respectively. *β*_2_*_i_* and Δ*n_i_* represent the dispersion and refractive index difference between two components for fiber with the length of *L_i_*. Δ*ω_x_*_/_*_y_* is the frequency separation between the central frequency *ω*_0_ and new-emerged *ω* of *u_x/y_* component.

**S5. Spectral confinement and sideband generation based on phase matching effect**

At a certain cavity position, the new frequencies generated between adjacent roundtrips interfere with each other. To give a clear demonstration, we assume that two lightwaves have the same intensity *I*_0_, and the interference results can be expressed as:

 (S7)

According to the Equation (S6), Δ*φ* changes with Δ*ω*, which results in the frequency-related interference curves. At the special Δ*ω*, Δ*φ* reaches the integral multiple of 2 π (i.e*.*, the phase matching condition) and results in the sharp spectral sidebands. The separations (Δ*ω_x_*_/_*_y_*) between sideband and central frequency are:

The theoretical results fully interpret the simulation and experiment results, such as the asymmetric spectrum and sidebands. In Figs. 1g and 1h of the manuscript, *m* is 1 and Δ*ω* is neglected when it exceeds the bandwidth of gain media.

**S6. DS in the YDF laser with a Lyot filter**

As the contrast experiment, we replace the polarization-insensitive isolator by a polarization-sensitive isolator to form a Lyot filter inside the same cavity. As shown in Fig. S4a, the modulation period of Lyot filter is ~1.7 nm for the PMF length of 1.5 m, which is too narrow to achieve mode locking in the fiber laser. Through diminishing the PMF length with a step of 0.05 m, we find that the modulation period is inversely proportional to PMF length (Fig. S4b), coinciding with the equation proposed by K. Özgören^4^.


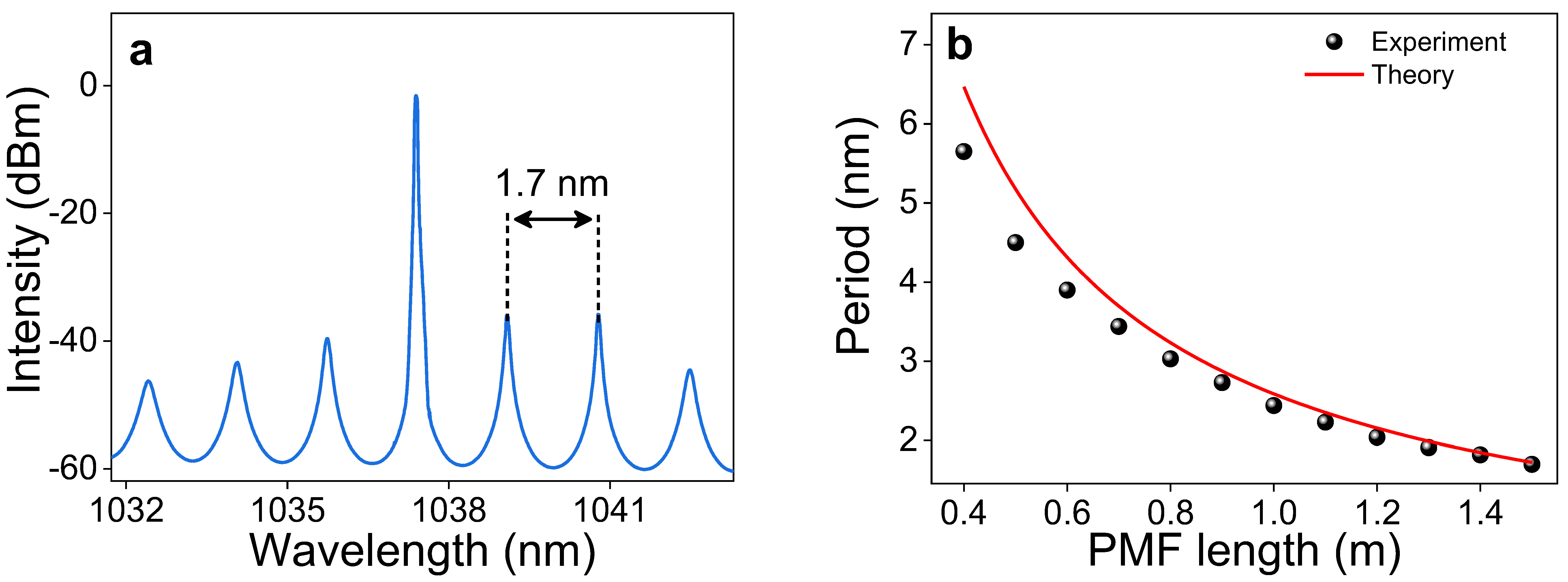


**Fig. S4 a** Laser spectrum for Lyot filter with 1.5 m PMF. **b** Modulation period of the Lyot filter versus PMF length.

The fiber laser is capable of emitting giant-chirp DS when the PMF length reaches ~0.4 m, as displayed in Figs. S5a and S5b. Based on the same simulation model, we numerically study the pulse evolution in the fiber laser including the Lyot filter, as shown in Figs. S5c and S5d. The measured (simulated) bandwidth and pulse duration are 1.05 nm (1.05 nm) and 39.6 ps (34.5 ps), giving the TBP of 11.64 (10.14), which indicates the giant-chirp property of DS that fundamentally differs from the near-chirp-free BMS.


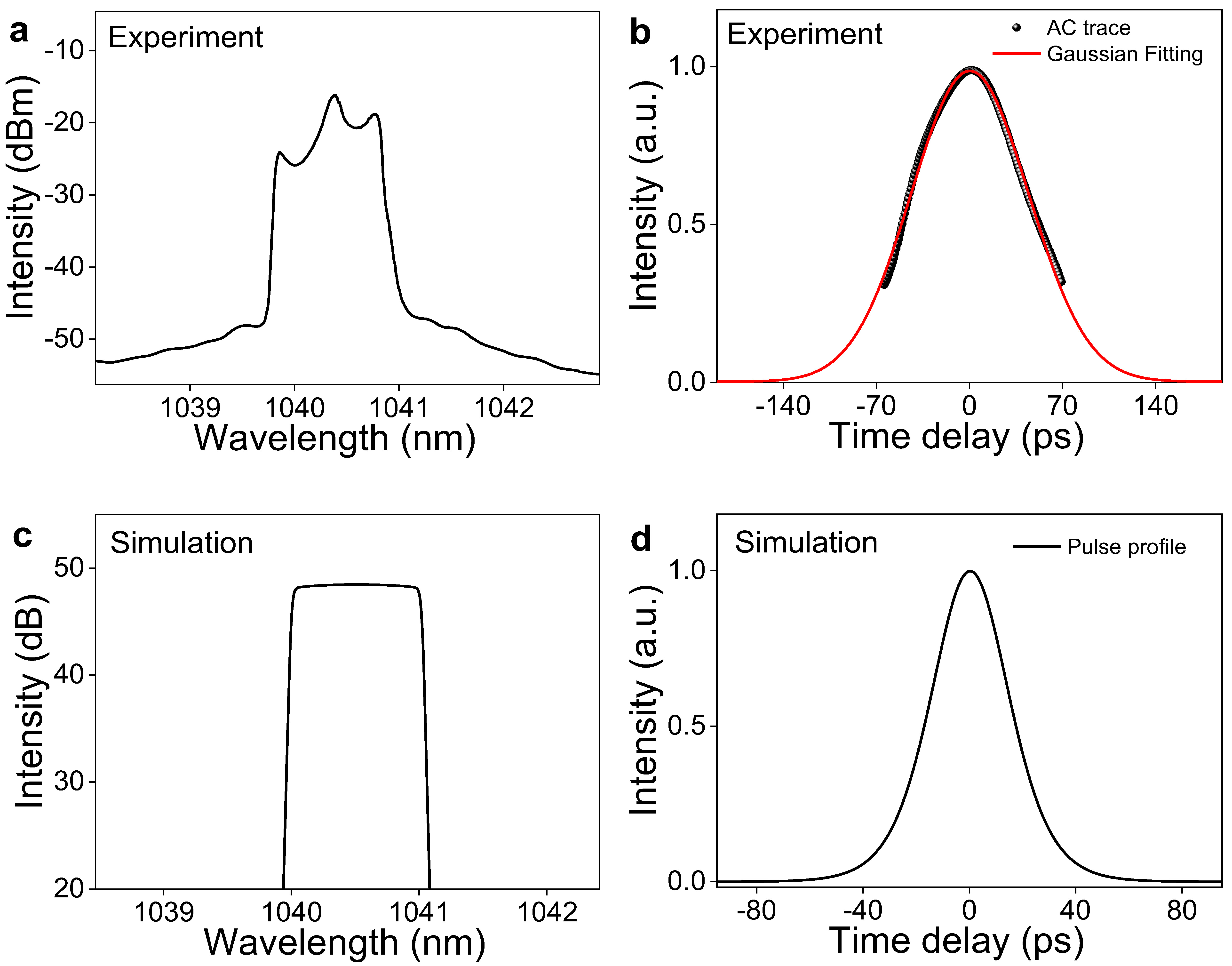


**Fig. S5 Giant-chirp DS obtained in the fiber laser with a Lyot filter.** **a** Measured spectrum, **b** autocorrelation trace and fit curve. **c** Simulated spectrum and **d** pulse profile.

**S7. BMS observed in normal-dispersion erbium-doped fiber lasers**

The same type of BMS can also be obtained in an erbium-doped fiber laser operating in normal-dispersion regime (single-mode fiber: 9.3 m; erbium-doped fiber: 15.3 m; polarization-maintaining fiber: 1 m). As displayed in Fig. S6a, the spectra of BMS display sidebands originated from the phase matching effect, and the retrieved pulse profiles and phases in Fig. S6b indicate the near-chirp-free property of the BMS. In this case, the TBP of BMS and each orthogonal-polarized component are ~0.529 (BMS), ~0.305 (*u_x_*) and ~0.302 (*u_y_*), respectively.


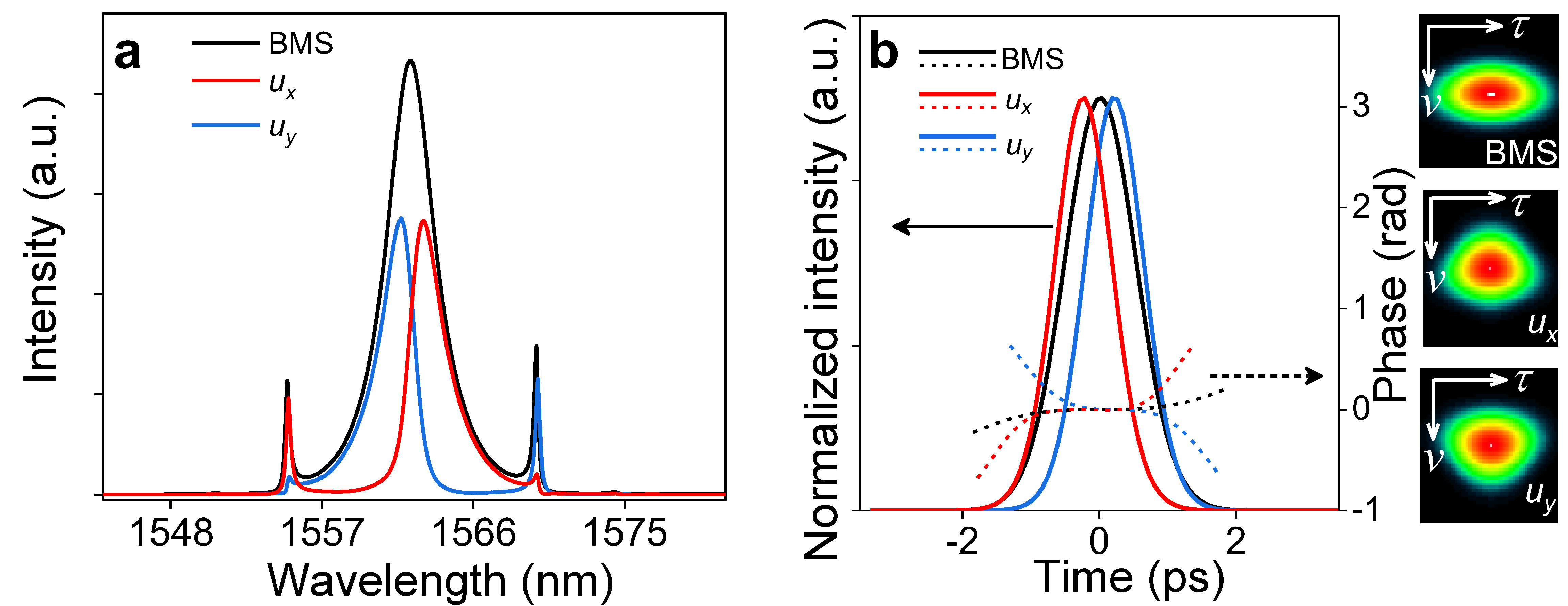


**Fig. S6 BMSs generated from a net-normal-dispersion erbium-doped fiber laser. a** Spectra and **b** retrieved pulse profiles, and FROG spectrogram: BMS (black curve), *u_x_* component (red curve), and *u_y_* component (blue curve).

**Supplementary Videos:**

**Video S1. Switching of near-chirp-free BMS and giant-chirp DS in ytterbium-doped fiber laser.**

**Video S2. Switching of near-chirp-free BMS and giant-chirp DS in erbium-doped fiber laser.**

**Supplementary References**

1 Wise, F. W., Chong, A. & Renninger, W. H. High-energy femtosecond fiber lasers based on pulse propagation at normal dispersion. *Laser & Photonics Review* **2**, 58-73 (2008).

2 Chong, A., Renninger, W. H. & Wise, F. W. Environmentally stable all-normal-dispersion femtosecond fiber laser. *Optics Letters* **33**, 1071-1073 (2008).

3 Grelu, P. & Akhmediev, N. Dissipative solitons for mode-locked lasers. *Nature Photonics* **6**, 84-92 (2012).

4 Ozgoren, K. & Ilday, F. Ö. All-fiber all-normal dispersion laser with a fiber-based Lyot filter. *Optics Letters* **35**, 1296-1298 (2010).
